# An Online Measurement Approach to Monitor the Deposition of Diesel Exhaust Particles on Lung Cells In Vitro

**DOI:** 10.1021/acs.est.4c08426

**Published:** 2025-06-16

**Authors:** Ruiwen He, Olivier Schaub, Christoph Geers, Aura Maria Moreno-Echeverri, Gowsinth Gunasingam, Sandor Balog, Maik Schultheiß, Bastian Gutmann, Tobias Krebs, Alke Petri-Fink, Barbara Rothen-Rutishauser

**Affiliations:** † Adolphe Merkle Institute, 311305 University of Fribourg, Chemin des Verdiers 4, 1700 Fribourg, Switzerland; ‡ Chemistry Department, 27211 University of Fribourg, Chemin du Musée 9, 1700 Fribourg, Switzerland; § NanoLockin GmbH, Route de la Fonderie 2, c/o Colab Fribourg, 1700 Fribourg, Switzerland; ∥ Vitrocell Systems GmbH, Fabrik Sonntag 3, 79183 Waldkirch, Germany

**Keywords:** DEPs, lock-in thermography, online particle monitoring, in vitro lung cell exposure

## Abstract

Diesel exhaust particles (DEPs) can deposit onto the respiratory epithelial surface upon inhalation. The quantification of DEP deposition on cells is important for understanding dose–response relationships within tissues. However, continuous, nondestructive methods for monitoring DEP deposition on cells remain challenging. We investigated a miniaturized thermosensitive detection system (CalorQuanti) and integrated it into the VITROCELL Cloud Alpha system, the so-called Cloud Alpha/CalorQuanti, to combine cell exposure to particles at the air–liquid interface (ALI) and online particle monitoring on cells. The Cloud Alpha/CalorQuanti demonstrates uniform particle deposition, providing the basis for online monitoring of particles on cells. With low detection limits (93 to 415 ng/cm^2^) for gold nanoparticles (AuNPs, as test material) and DEPs, the device showed strong positive relationships (*R* ^2^ ≥ 0.95) between particle concentrations measured and thermal signal intensities generated on cells under dry and humid conditions. It also possesses the versatility to optimize online particle detection on cells by adjusting measurement parameters to balance sensitivity and phototoxicity. The Cloud Alpha/CalorQuanti allows exposure to aerosolized DEPs at ALI while providing sensitive monitoring and quantification of DEP deposition on cells. This device is useful for monitoring combustion-derived carbon-based particle exposure on cells following laboratory simulation and field exposures, advancing environmental health research.

## Graphical abstract

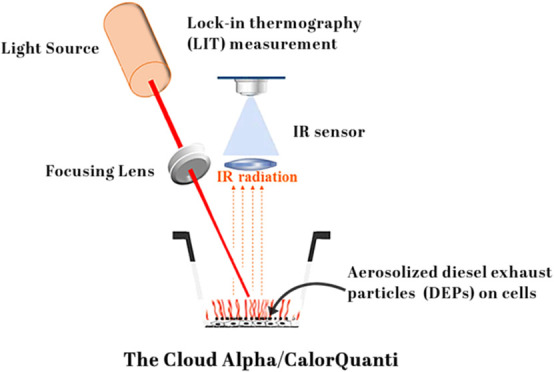

## 1 Introduction

Traffic-related air pollution is a major environmental health concern worldwide, in which particles emitted from vehicle exhaust, road dust, and tire wear are the main contributors.^1–3^ Diesel exhaust particles (DEPs) produced by diesel engines are of particular concern due to their complex mixtures of different chemical components and small sizes.^4^ DEPs are present in the atmosphere in the trimodal size distribution, with aerodynamic diameters ranging from 10 nm to over 1 μm.^5^ The small size range makes DEPs respirable and likely to deposit on the respiratory epithelial surface,^6^ thereby posing risks to human health. Inhaled DEPs are potentially inflammatory and carcinogenic.^7,8^

There has been a growing interest in assessing the inhalation toxicity of particles, including DEPs, on human health through *in vitro* exposure of lung cells to particles in past years.^9–12^ To closely mimic *in vivo* conditions, lung cells are grown on the permeable membrane with the apical side exposed to air and the basal side in contact with the medium to absorb nutrients, known as air–liquid interface (ALI) culture. Cells under ALI culture can be directly exposed to aerosols, reflecting realistic human inhalation scenarios.^13–16^ Various aerosol exposure systems, including some in-house versions and commercial versions such as CULTEX Radial Flow System, PreciseInhale system, and VITROCELL Cloud exposure system, have been developed for ALI exposure to particles via diffusion/sedimentation or airflow-delivery approaches.^14^ Among these exposure systems, the Cloud exposure system has been widely used in recent years, which allows the generated particle droplets to be evenly sedimented on cells for exposure under ALI conditions.^17^ The quantification of DEP deposition on cells is important for the dose–response relationships within cells,^18^ efforts have been made to quantify and visualize particle deposition in the exposure experiments, such as installing a quartz crystal microbalance (QCM)^19^ to measure the mass concentration or positioning a transmission electron microscopy (TEM) grid^20^ to analyze size and morphology of deposited particles. Additionally, methods including atomic force microscopy,^21^ Raman microspectroscopy,^22^ and TEM^23^ have also been used to visualize DEPs deposited on cells. In addition to these methods, there is a demand for a user-friendly and nondestructive method that can monitor DEPs on cells online during exposure.

Notably, nondestructive particle detection methods using light illumination have been developed in recent years.^24–26^ Lock-in thermography (LIT) stands out as a promising technique that operates by subjecting particles to periodic amplitude-modulated stimulation under light illumination while simultaneously recording changes in the particle surface temperature using an infrared sensor/camera.^24,26,27^ Through analysis of temperature oscillations, LIT can efficiently filter out background noise and detect temperature differences as small as 0.001 K.^28^ This is a continuous monitoring technique that allows uninterrupted, real-time collection and analysis of particle information. The remarkable capability sparks interest across various fields, including investigations into aerosol deposition, nanoparticle behaviors, and cell-particle interaction.^29^ This method has been used to monitor thermoresponsive particles such as gold nanoparticles (AuNPs) and carbon nanotubes.^27,30^

The primary objective of this study was to develop a user-friendly and versatile platform for noninvasive online monitoring of DEPs deposited on lung cells during ALI exposure. To achieve this goal, we integrated a miniaturized LIT system (called CalorQuanti) into the VITROCELL Cloud Alpha system to achieve the required technical functionalities. Our experiments start with performance testing of the newly developed setup, the so-called Cloud Alpha/CalorQuanti, using AuNPs as the test material and A549 cells representing human alveolar epithelial cells. This evaluation included the assessment of particle distribution in the device base after aerosolization, as well as the sensitivity and stability of the device in monitoring particles deposited on cells. On the basis of the obtained results, DEP monitoring was performed in the Cloud Alpha/CalorQuanti with A549 cells under both fixed (relatively dry) and culture (high humidity) conditions, with parameter optimization carried out as necessary.

## 2 Methods

### 2.1 Human Alveolar Epithelial Cell Culture

The A549 cell line, derived from adenocarcinoma human alveolar basal epithelial cells,^31^ was purchased from the American Type Cell Culture Collection (ATCC). Cells were cultured and maintained in culture flasks with Roswell Park Memorial Institute (RPMI) 1640 culture medium supplemented with 10% fetal bovine serum, 1% penicillin/streptomycin, and 1% l-glutamine in an incubator with 5% CO_2_ at 37 °C. The cell culture medium was changed every 3–4 days. Upon reaching approximately 80% confluency within the culture flask, A549 cells (passage number: 6–16) were enzymatically detached using 0.05% trypsin-EDTA. Subsequently, 1 mL of A549 cell suspension was seeded on the apical side of 6-well inserts (4.67 cm^2^, 0.4 μm pore membranes, Falcon, Switzerland) at a density of 250,000 cells/mL. These inserts with cells were positioned in 6-well plates with 2.0 mL culture medium on the basal side and cultured for 4 days in an incubator to reach confluence. Cells were then either fixed using 4% paraformaldehyde (PFA, Sigma-Aldrich, Switzerland) in PBS or cultured under ALI conditions depending on experimental purposes (described in Section 2.4). ALI culture of A549 cells was performed according to the PATROLS standard operation protocol (SOP).^32^ Following the 4-day culture, the apical medium was removed, the basal medium was refreshed, and A549 cells were apically exposed to air to obtain the ALI conditions. Cells were cultured at the ALI for 24 h in the incubator at 37 °C and 5% CO_2_ before exposure. Unless otherwise stated, all culture media and supplements used in this study were purchased from Gibco and Thermo Fisher Scientific (Switzerland).

### 2.2 Preparation and Characterization of Particle Suspension

AuNPs (50 nm, 0.14 mM) were synthesized following the Turkevich method^33^ from the 15 nm seed and then followed by the Brown method,^34,35^ as previously described.^36^ Details on the synthesis of AuNPs can be found in the Supporting Information. Standardized DEPs (Standard Reference Material 2975), representing traffic-related air pollution, were obtained from the National Institute of Standards and Technology (NIST) and dispersed in sterile Milli-Q (MQ) water (resistivity 18.2 MΩ cm, Sartorius, Germany) following the NANOGENOTOX dispersion protocol.^37^ Ultraviolet–visible spectroscopy (UV–vis) spectra of AuNP and DEP suspensions were recorded using a spectrophotometer (model V-670, Jasco) at 25 °C with 10 mm path-length disposable UV cuvettes (Sarstedt, Germany). Hydrodynamic diameters of AuNPs and DEPs in suspensions were determined by performing dynamic light scattering (DLS) in MQ water as a dilute suspension using a particle analyzer (model Litesizer 500, Anton Paar, Graz, Austria) with a 658 nm laser at a scattering angle of 175° and advanced cumulant model. To visualize AuNPs and DEPs, the diluted particle suspension in MQ water was drop-cast onto a 300-mesh carbon membrane-coated copper grid (Electron Microscopy Sciences) and air-dried overnight at room temperature before examination via TEM (model FEI Tecnai G2 Spirit, Thermo Fisher Scientific) equipped with a 120 kV LaB6 emitter and a 2048 × 2048 pixel wide-angle Veleta camera (Olympus, Japan). MQ water served as the control or reference for background correction throughout all characterizations.

### 2.3 Building the Cloud Alpha/CalorQuanti

The Cloud Alpha/CalorQuanti (Figure 1) was developed for cell exposure to particles and online particle monitoring on cells, consisting of the VITROCELL Cloud Alpha 6 (Figure 1A, VITROCELL Systems GmbH, Germany) and the miniaturized LIT system CalorQuanti (Figure 1B, NanoLockin GmbH, Switzerland). The Cloud Alpha system is comprised of an aerosol exposure chamber, a base module with five well positions for placing cell culture inserts, and a nebulizer (4.0–6.0 μm droplet size) for aerosolizing particles. In addition, a QCM is installed in the base module to determine the mass concentration of particles deposited. The CalorQuanti has been designed for particle detection using a thermosensitive method based on the lock-in detection principle,^29^ as previously reported.^24,26^ The CalorQuanti can emit the excitation light at different wavelengths and frequencies to generate heat in the light-absorbing particles deposited on cells in the inset at position 1. An infrared sensor (model PIP-UC-LS, VIGO Photonics, Poland) mounted on the exposure chamber vertically at position 1 was used to simultaneously record the full-frame thermal signal generated by particles on cells. The collected thermal information was processed to compute amplitude maps according to the lock-in detection principle, then transferred to digital output via a custom Python-based integrated development environment (IDE) (Spyder IDE, 5.3.3; Python 3.8.10), thereby achieving continuous, nondestructive, online monitoring of particles on cells. A schematic diagram of thermal information processing and the workflow of this device with representative images of data generation, including time domain signal and frequency domain spectrum, are shown in Figures S1 and S2. A video showing the Cloud Alpha/CalorQuanti in action is also provided in the Supporting Information.

**Figure 1:**
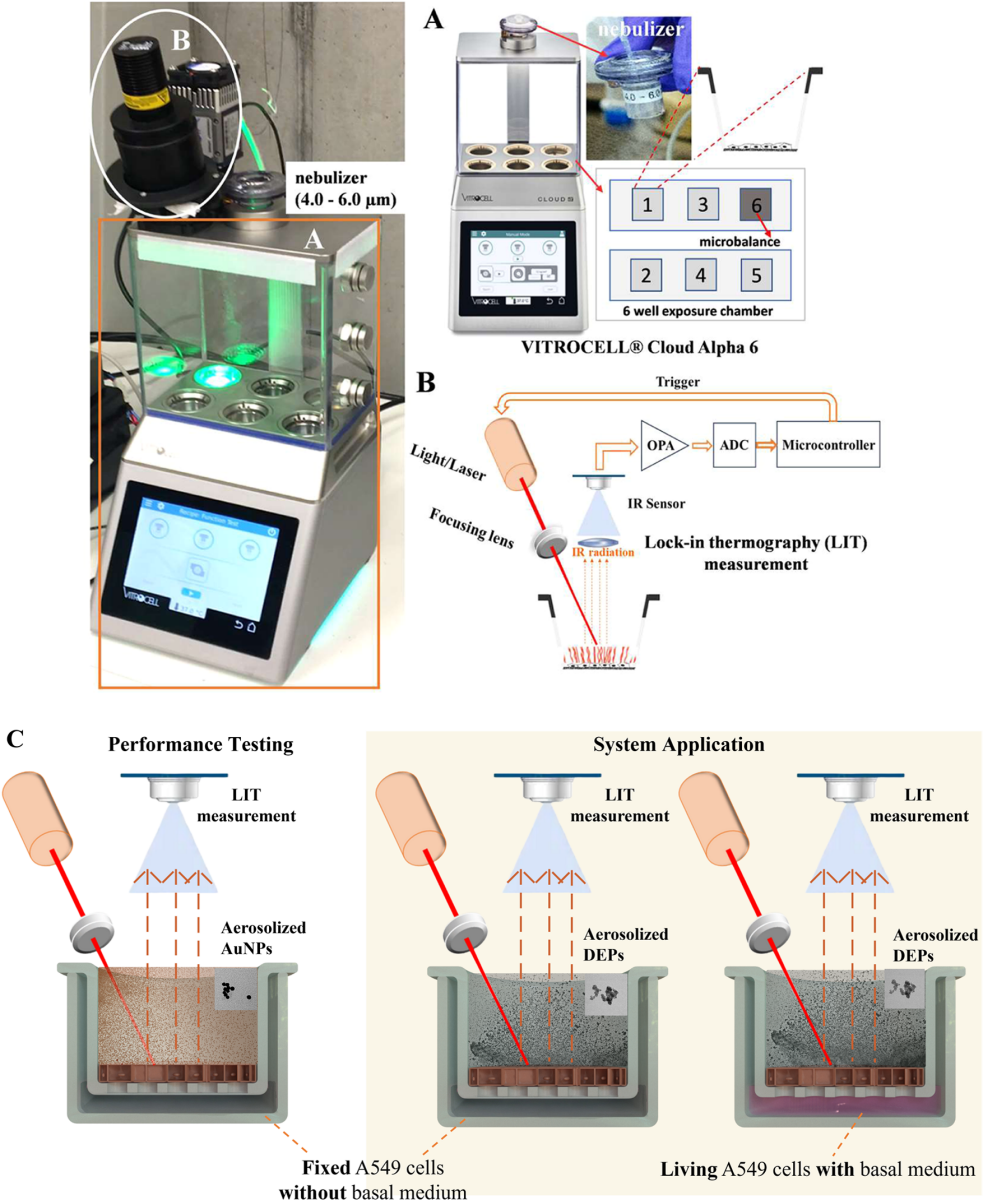
Cloud Alpha (A)/CalorQuanti (B) combines cell exposure to particles and online particle monitoring on cells. With the Cloud Alpha system (A), particles are aerosolized via a nebulizer (4.0–6.0 μm) onto cells grown on inserts positioned in module wells (positions 1–5) of the exposure chamber. The deposited particle mass was determined via a QCM installed at position 6. Then the CalorQuanti (B) measures thermal signal intensities of deposited particles on cells by using modulated light illumination to heat particles, a technique known as lock-in thermography (LIT). IR: infrared; OPA: operational amplifier; ADC: analog to digital converter. (C) Experimental procedures illustrate performance testing of the Cloud Alpha/CalorQuanti using AuNPs, a practical LIT-detectable reference, with fixed A549 cells in the absence of culture medium. Next, the Cloud Alpha/CalorQuanti was used to monitor standardized DEPs during cell exposure in two experimental setups: fixed A549 cells without culture medium (dry conditions) and living A549 cells under ALI culture with the basal medium for particle exposure (humid conditions). Vitrocell logo reproduced with permission from Vitrocell Systems GmbH.

### 2.4 Experimental Procedures

Experimental procedures are illustrated in Figure 1C. The performance testing of the Cloud Alpha/CalorQuanti was done first using AuNPs, a practical LIT-detectable reference.^30^ These tests were conducted on fixed A549 cells in the absence of culture medium from a reproducible and practical point of view. Specifically, particle distribution on cells after exposure was assessed, and the sensitivity and operational stability of the device in particle monitoring over consecutive days were evaluated. Next, the Cloud Alpha/CalorQuanti was used to monitor standardized DEPs during cell exposure in two experimental setups:^1^ fixed A549 cells in the absence of culture medium (dry conditions). This setup reflects scenarios of particle collection and deposition on solid support (*e.g.*, filters or membranes) during laboratory simulation or field exposure; (2) living A549 cells under ALI culture with the basal medium for particle exposure and deposition on the apical cell side (humid conditions). This setup is usually used in aerosol toxicity testing to mimic the physiologically relevant inhalation scenario. In both conditions, measurement parameters were optimized, and correlations between DEP mass concentrations and the generated thermal signal intensities were established.

### 2.5 Performance Testing of the Cloud Alpha/CalorQuanti

Cell exposure to AuNPs was performed in the Cloud Alpha/CalorQuanti according to the PATROLS SOP.^38^ Five inserts with fixed A549 cells were placed in the wells of the base module (position 1–5, Figure 1A) without the addition of basal medium. Subsequently, 200 μL of AuNP suspension was aerosolized, generating a dense cloud of AuNP droplets in the exposure chamber. Within minutes of droplet sedimentation, particles deposited on the base module, where inserts were positioned, and the mass concentration of deposited particles can be recorded by a QCM. We repeatedly aerosolized AuNP suspension in the exposure chamber to achieve a wide deposited concentration range of AuNPs (0–646 ng/cm^2^). Between each aerosolization, the thermal signal intensity of AuNPs on cells at position 1 was continuously measured 10 times under 525 nm light-emitting diode (LED) light with a stimulation frequency of 1 Hz. Each measurement lasted 1 min (i.e., 60 cycles), resulting in a total continuous measurement duration of 10 min per concentration. The relationship between mass concentrations of AuNPs deposited on cells and corresponding thermal signal intensities generated was established. In addition, under the same light conditions, thermal signal intensities of AuNPs at 147 and 473 ng/cm^2^ on cells in well positions 2–5 were determined to assess particle distribution at the base of the device. AuNPs at 473 ng/cm^2^ on cells and negative control (0 ng/cm^2^, exposure to MQ water via aerosolization) were measured over three consecutive days to assess the device stability in particle monitoring.

To monitor AuNP distribution on the cell layer, thermal imaging recordings, as previously described,^30^ were conducted for AuNPs at 0, 147, 260, and 473 ng/cm^2^. Inserts were placed on a light homogenizing glass rod (model N-BK7, Edmund Optics) between an LED panel and an infrared camera (model Onca-MWIR-InSb-320, XenICs, Belgium). Recordings were performed under a 525 nm light with a stimulation frequency of 1 Hz and an applied current of 150 mA (power density: 45.3 mW). Each measurement lasted 1 min (i.e., 60 cycles), and the same settings were maintained for all tested samples. To visualize AuNPs deposited on cells, insert membranes were cut and placed on coverslips for examination by the enhanced darkfield hyperspectral imaging (DF-HSI, Cytoviva Inc., Auburn) at a 50 × objective lens.

### 2.6 Application of Cloud Alpha/CalorQuanti in Monitoring DEPs on Cells

#### 2.6.1 Cell Exposure to DEPs and DEP Monitoring on Fixed A549 Cells

Following the 2.5 procedure, DEPs were repeatedly aerosolized in the exposure chamber to achieve deposited concentrations from 0 to 450 ng/cm^2^ on fixed A549 cells without the basal medium. LIT measurements were conducted between each aerosolization to monitor DEPs on cells under a 525 nm light, with parameters (frequency and measurement duration) optimized for effective monitoring. Thermal signals of DEPs at each concentration were continuously measured 10 times and each measurement lasted 1 min, resulting in a total continuous measurement duration of 10 min per concentration. The relationship between DEP concentrations on cells and generated thermal signal intensities was established. Thermal imaging recordings were performed for DEPs at 0, 240, and 440 ng/cm^2^ on cells. To visualize DEP deposition after aerosolization, a TEM grid sampler, as previously described,^20^ was placed in the base module well (position 5) before DEP aerosolization and then collected after DEP sedimentation for TEM.

#### 2.6.2 Effect of the Basal Medium on DEP Detection

During ALI culture and exposure, cell culture medium added into the basal compartment for nutrient supply may cause thermal absorption during LIT measurements. The effect of the basal medium on DEP detection with LIT was assessed. Cell culture medium (3 mL) was added into each module well on the basal side of inserts with fixed A549 cells to simulate ALI culture conditions for the cells. The exposure chamber humidity was recorded before and after adding medium via a relative humidity (RH) and temperature controller (±1.5% RH, Vitrocell Systems GmbH, Germany). To enhance the generated thermal signal intensity, a 637 nm red laser diode (model HL63283HD, Ushio, Japan) was used as an alternative light source for monitoring DEPs on cells, in comparison with the 525 nm LED. LIT parameters, including frequency and measurement duration (*i.e.*, cycles), were optimized for effective DEP monitoring.

#### 2.6.3 Cell Exposure to DEPs and DEP Monitoring in Living A549 Cells

Inserts with the living A549 cells were placed in wells of the base module with 3 mL of basal medium. By using living cells, we could examine the effects of cell culture parameters, *i.e.*, temperature and humidity, on thermal signal intensity and optimize measurement parameters to balance signal strength and phototoxicity. Following the exposure procedure in section 2.5, DEPs were repeatedly aerosolized to achieve a mass concentration range (0–1010 ng/cm^2^) deposited on cells. Between each aerosolization, LIT measurements with laser were performed 5 times for monitoring DEPs on cells under the optimized parameters. Each measurement lasted 3 min, resulting in a 15 min continuous measurement duration per concentration. The relationship between DEP mass concentrations in living cells and generated thermal signal intensities was established.

#### 2.6.4 Phototoxic Effects of LIT Measurements with Laser on Cells

LIT measurements with laser were respectively performed one, three, and five times in living A549 cells at ALI culture conditions under the optimized parameters. This procedure represents exposure scenarios, including repeated LIT measurements after a single exposure and successive exposures with a single LIT measurement in between. To assess the phototoxic effects, inserts with cells were then replaced into new 6-well cell culture plates with 2 mL fresh culture medium, and postincubated for 24 h before phototoxicity analysis.

#### 2.6.5 Cytotoxicity and Cell Morphology

Lactate dehydrogenase (LDH) leakage is an indicator of cytotoxicity. LDH assay and immunofluorescence staining of cells were performed 24 h after LIT measurements to assess cytotoxicity and cell membrane integrity. Details are provided in the Supporting Information.

### 2.7 Data Analysis

Values of LIT measurements were from 5 to 10 independent replicates. Values of LDH release were from three biologically independent replicates, with two parallel samples in each experiment. Data analysis was performed using the GraphPad Prism (version 9.5.1, GraphPad Software Inc.). Statistical analysis between groups used parametric one-way analysis of variance (ANOVA) with Tukey’s test, and a significant difference was defined as *p* < 0.05. The piece-wise linear regression curves were obtained via ordinary least-squares using Mathematica (Model Mathematica 14, Wolfram) to interpret the experimental data, which consisted of (a) an apparent baseline, where the signal detected was constant and independent of concentration, and (b) a linear range, where the signal detected showed evident proportionality with concentration. The limits of detection (LOD) and quantification (LOQ) were estimated according to the approach of Hubaux and Vos, using a 95% confidence level.^39^ We took several quality control measures in this study: AuNP and DEP suspensions were freshly prepared before exposures and aerosolized via a large droplet size nebulizer (4.0–6.0 μm) relative to particle size distribution to reduce particle agglomeration. Suspension quality, including particle size, morphology, and dispersion state, was validated prior to use. After aerosolization, particle deposition patterns were visualized. For experimental calibration and validation, negative control was measured before, during, and after each experiment to account for baseline drift and environmental noise. Particle concentration-thermal signal calibration curves were established across particle types and exposure conditions (*e.g.*, dry and humidity) to serve as reference standards for data interpretation. Details on the treatment of outliers and baseline measurements can be found in the Supporting Information.

## 3 Results

### 3.1 Characterization of AuNPs and DEPs in Milli-Q (MQ) Water

AuNP and DEP suspensions were prepared in MQ water at concentrations of 128 and 80 μg/mL, respectively. Hydrodynamic diameters of AuNPs and DEPs, assessed through DLS, were 54 and 189 nm, respectively. The morphology of AuNPs and DEPs was assessed using TEM, showing that AuNPs were spherical, whereas DEPs exhibited heterogeneous shapes. UV–vis spectra spanning 350–750 nm revealed that AuNPs exhibited an absorption peak at 525 nm, while DEPs displayed a decrease in absorption (Figure S3).

### 3.2 Performance Testing of the Cloud Alpha/CalorQuanti Using AuNPs

AuNPs were used to test the Cloud Alpha/CalorQuanti performance with fixed A549 cells in inserts without adding the basal culture medium. A positive relationship (*R* ^2^ = 0.95) was established between AuNP concentrations (12–646 ng/cm^2^) on the cell layer measured by a QCM and generated thermal signal intensities, with the LOD for AuNPs determined to be 93 ng/cm^2^ (Figure 2A). Although several areas showed slightly elevated thermal signals likely from localized deposited particle clusters, the corresponding thermal images showed the relatively homogeneous signal intensity on cells in each insert, increasing with AuNP concentrations (Figure 2B). The detected thermal signal intensities of AuNPs at 147 and 473 ng/cm^2^ on cells across inserts from different module wells (position 1–5) were comparable and the signal intensity remained consistent between three consecutive days under the same LIT measurement conditions (Figure S4). Additionally, AuNPs at 473 ng/cm^2^ on cells were visualized by DF-HSI (Figure 2C), showing well-distributed AuNPs on the cell layer.

**Figure 2:**
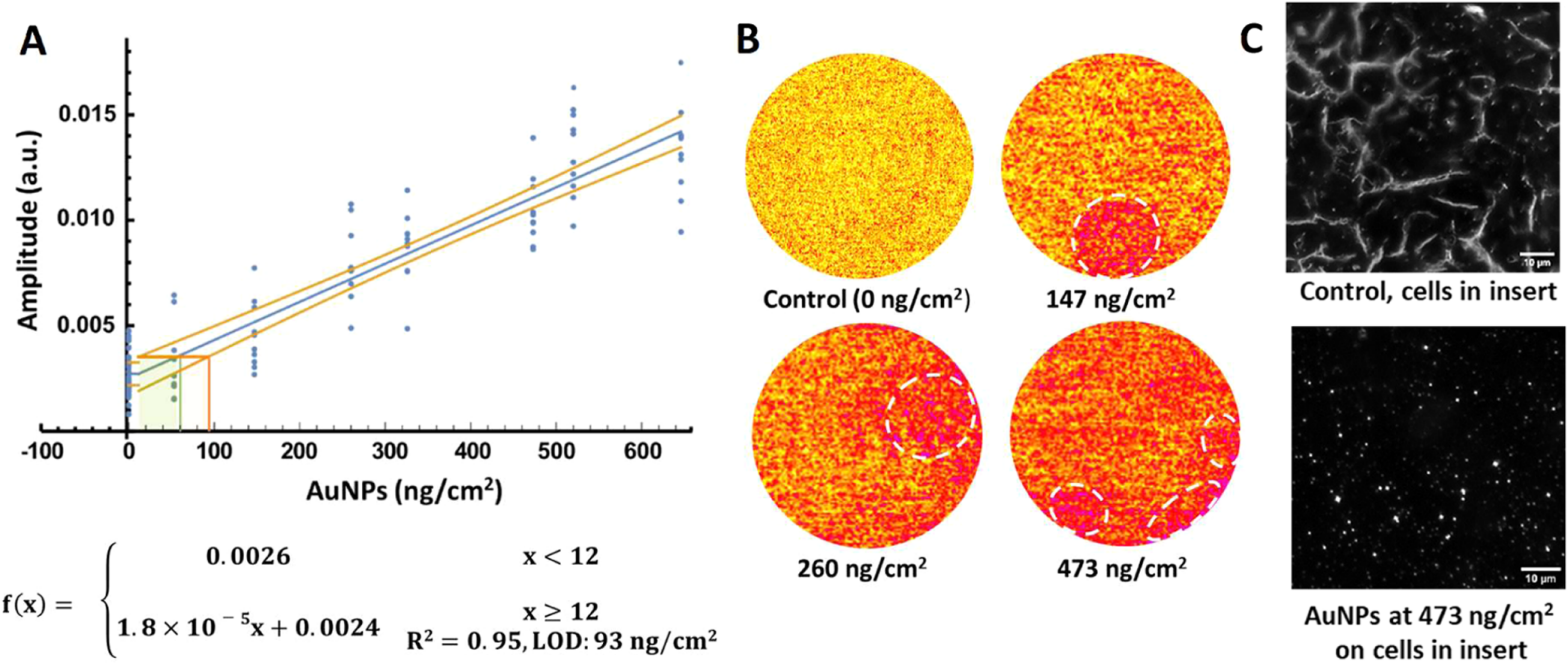
LIT measurements for AuNPs on fixed A549 cells without basal medium using light with a wavelength of 525 nm at a stimulation frequency of 1 Hz. (A) Relationship between QCM-measured AuNP concentrations (0–646 ng/cm^2^) on cells and thermal signal intensities; (B) thermal imaging recordings at selected concentrations (0, 147, 260, and 473 ng/cm^2^), with highlighted areas reflecting relatively higher thermal signatures. LOD: limit of detection; (C) Dark-field images of AuNPs at 0 and 473 ng/cm^2^ on cells. Scattered dots (in A) indicate values obtained from ≥10 continuous independent measurements. Each measurement lasted 1 min (*i.e.*, 60 cycles). The raw numerical values for each dot can be found in Supporting Information Table S1. Scale bar in C: 10 μm.

### 3.3 Cloud Alpha/CalorQuanti for Cell Exposure to DEPs and DEP Monitoring

DEP suspension (80 μg/mL) was repeatedly aerosolized in the exposure chamber to achieve the deposited concentrations ranging from 0 to 450 ng/cm^2^ measured by a QCM, which were selectively visualized through TEM. TEM images showed the almost uniform distribution of DEPs at 240 and 440 ng/cm^2^, with several particle clusters, after sedimentation on the base module of the Cloud Alpha/CalorQuanti, and the higher particle density was observed with increasing mass concentrations. Parameter optimization of LIT measurements for monitoring DEPs on the cell layer (fixed cells) was performed with control (0 ng/cm^2^) and exposed cell sample (440 ng/cm^2^) without basal medium under a light wavelength of 525 nm. According to Figure S5, LIT parameters were set at a stimulation frequency of 1 Hz, and each measurement lasted 1 min (*i.e.*, 60 cycles). Under the optimized parameters, thermal images of DEPs at 240 and 440 ng/cm^2^ on cells were generated (Figure 3A). While the overall thermal signal intensity map appeared relatively homogeneous, certain areas showed slightly higher thermal signatures, which are likely due to the deposited particle clusters. The increasing signal intensities were also detected corresponding to higher DEP concentrations, consistent with the observation from TEM images. By testing more exposed cell samples (Figure 3B), a robust positive relationship (*R* ^2^ = 0.98) was established between DEP concentrations (62–450 ng/cm^2^) measured by a QCM on cells and generated thermal signal intensities, with a low LOD (93 ng/cm^2^) for DEPs. Significant differences (*p* < 0.05) were shown between intensities of the control (0 ng/cm^2^) and DEPs ≥ 130 ng/cm^2^, comparable to the LOD level, deposited on the fixed cell layer.

**Figure 3:**
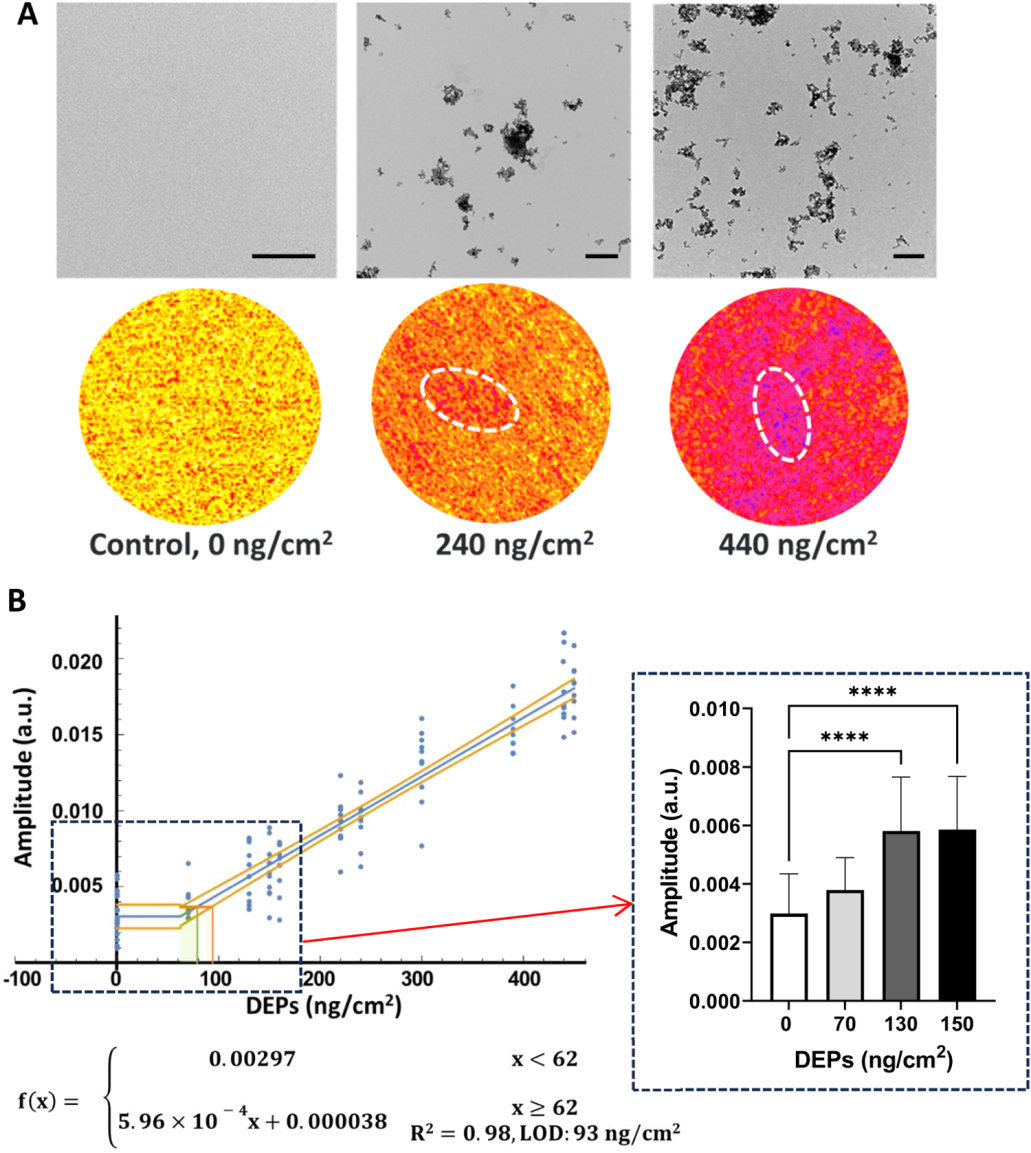
LIT measurements were performed for DEPs (0 to 450 ng/cm^2^) on fixed A549 cells in inserts without basal medium under a light wavelength of 525 nm with a stimulation frequency of 1 Hz. (A) TEM images of the deposited DEPs at 240 and 440 ng/cm^2^ on the TEM grid placed in the base module of the Cloud Alpha/CalorQuanti and the corresponding thermal imaging recordings of DEPs on the fixed cells, with highlighted areas reflecting relatively higher thermal signatures; (B) The relationship between QCM-measured DEP concentrations on cells and generated thermal signal intensities. LOD: the limit of detection. ****: *p* < 0.0001. Scale bar in (A): 2 μm for control and 500 nm for DEP samples. Scattered dots in (B) indicate values obtained from ≥ 10 continuous independent measurements. Each measurement lasted 1 min (i.e., 60 cycles). The raw numerical values for each dot can be found in Supporting Information Table S1.

### 3.4 Detection of DEPs on Cells under ALI Exposure Conditions

The relative humidity was around 60% without the medium and increased to >95% after the addition of basal medium to the module wells containing the cell inserts. The addition of the basal medium resulted in thermal signal intensities of DEPs (440 ng/cm^2^) on cells at the control level under a light wavelength of 525 nm at stimulation frequencies ranging from 0.125 to 8 Hz (Figure S6A). In comparison, the detected thermal signal intensity significantly improved when using a laser wavelength of 637 nm. Although high-humidity conditions (with the basal medium) can largely reduce thermal signal intensity compared to dry conditions, the relative data variation is comparable between humid (16%) and dry conditions (19%) under the 637 nm laser (Figure S6B), suggesting that humidity may not affect the thermal signal stability. As such, further LIT measurements to monitor DEPs in living cells under ALI culture conditions were performed using a laser wavelength of 637 nm.

The optimal thermal response for DEP detection occurred at a stimulation frequency of 0.25 Hz among the frequencies tested with a laser wavelength of 637 nm. However, to gather more data within the same time frame, we selected a frequency of 4 Hz for further LIT measurements, which also induced a relatively high thermal response, with each measurement lasting 3 min (i.e., 720 cycles, Figure S6A). Under the optimized LIT parameters, we measured thermal signal intensities of DEPs ranging from 0 to 1010 ng/cm^2^ in living A549 cells under ALI exposure conditions. A strong positive relationship (*R* ^2^ = 0.96) was established between concentrations of DEPs (345 to 1010 ng/cm^2^) deposited on cells and generated thermal signals, with the LOD for DEPs in living A549 cells determined to be 415 ng/cm^2^ (Figure 4A). Significant differences (*p* < 0.05) in thermal signal intensities were shown between control (0 ng/cm^2^) and DEPs ≥ 457 ng/cm^2^, comparable to the LOD level, in the living cells.

**Figure 4:**
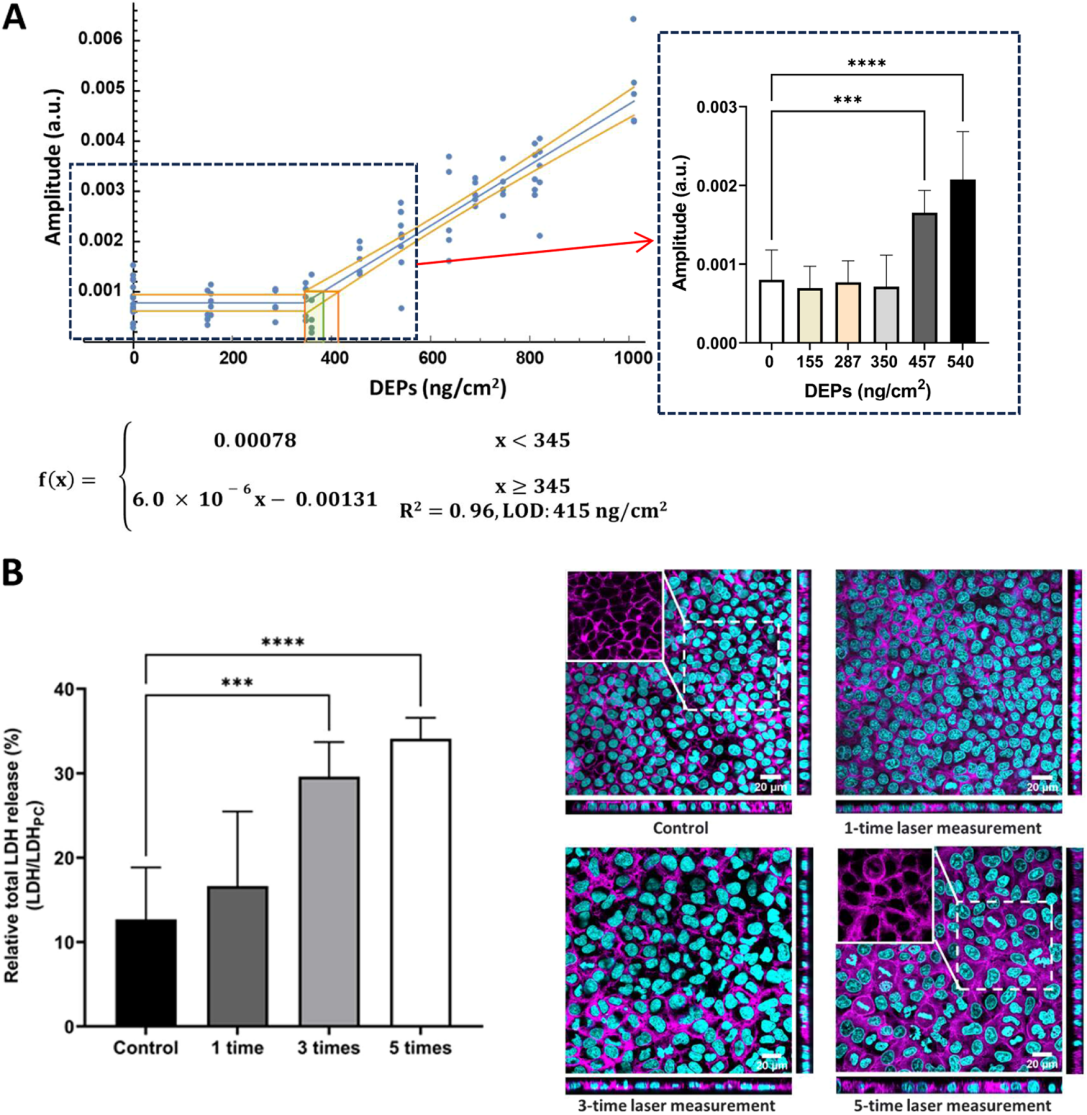
(A) Relationship between DEP concentrations (0 to 1010 ng/cm^2^) measured by a QCM on cells and the generated thermal signal intensities. LIT measurements were performed for monitoring DEPs in living cells in inserts at the ALI conditions (with basal medium) at a laser wavelength of 637 nm with a stimulation frequency of 4 Hz. LOD represents the limit of detection. Scattered dots indicate values obtained from ≥ 5 continuous independent measurements. Each measurement lasted 3 min (i.e., 720 cycles). The raw numerical values for each dot can be found in Supporting Information Table S1. *** represents *p* < 0.001 and **** *p* < 0.0001. (B) Relative LDH release and confocal laser scanning microscopy (cLSM) images of the A549 cells 24 h after LIT measurements for one, three, and five times under a laser wavelength of 637 nm at 4 Hz. Each measurement lasted 3 min (*i.e.*, 720 cycles). Inserts with cells that were treated in the same way but without LIT measurements served as controls. As a positive control (PC), Triton X-100 (1%, v/v in medium) was applied apically for 24 h. Error bars indicate the standard deviation of 6 parallel cell samples from three independent experiments. *** represents *p* < 0.001 and **** *p* < 0.0001. Cyan represents nuclei, and magenta represents filamentous actin (F-actin), scale bars: 20 μm.

The potential phototoxic effects of LIT measurements with laser on cells were assessed 24 h after one, three, and five consecutive LIT measurements (Figure 4B). The relative LDH release in the negative controls is around 12%, reflecting no cytotoxicity. LDH release was at the control level (≈ 15%) following one-time LIT measurement but significantly increased (≈ 30%) after three and five repeated LIT measurements (Figure 4B). The confocal laser scanning microscopy images of A549 cells showed epithelial monolayer morphology was still intact after LIT measurement for one, three, and five times (Figure 4B). However, after five LIT cycles, F-actin staining appeared more dispersed and disorganized, with large gaps at cell borders, suggesting potential disruption of cell–cell adhesion.

### 3.5 Data Variation for LIT Measurements under Different Parameters

Baseline thermal signals were established using a negative control (0 ng/cm^2^) with at least five LIT measurements. The raw numerical values for each negative control measurement can be found in Table S1, ranging from 0.00248 ± 0.00098 to 0.00317 ± 0.00171 under dry conditions and from 0.00069 ± 0.000373 to 0.00097 ± 0.000385 under humid conditions. Importantly, our data analysis has not revealed any systematic correlation between baseline drift and extended experimental runs.

To assess the effects of setting parameters and particle concentrations on data reproducibility, the relative variation as the ratio of standard deviation to the mean was calculated across different conditions (Figures S5 and S7). The relative variation increased from 4 to 11% as frequency increased from 1 to 4 under 525 nm LED light. This is likely due to reduced thermal penetration depth at higher modulation frequencies, causing weaker temperature oscillations and a greater relative contribution of noise. No changes in the relative variation were observed for the used measurement duration (60s and 180s). Additionally, DEPs at 440 ng/cm^2^ on fixed cells generated a much stronger relative thermal signal under the 637 nm laser than under the 525 nm LED with the same measurement parameters. Laser Illumination introduced larger data variation (17 vs 4%), likely due to uneven illumination and higher output power fluctuation. In addition, negative controls and low-concentration particle samples showed a higher relative variation (>40%, Figure S7), which decreased to around 15% when the particle concentrations exceeded the LOD. This trend likely results from the lower thermal signals in negative controls and low-concentration samples, where background noise becomes more dominant, causing higher data variation.

### 3.6 Comparison of QCM and LIT Techniques

We have compared the QCM and LIT techniques in the Cloud exposure system (Table S2). QCM offers high-speed measurement with a broad dynamic range and a slightly higher variation (32%), making it well-suited for detecting rigid-layer deposits. However, it cannot quantify the mass of previously deposited particles on the layer and requires an exposure well for installation in the Cloud exposure system. In contrast, LIT provides nondestructive measurements, greater flexibility, and continuous monitoring to detect thermal signals of particles deposited on cells, without the need for an exposure well, making it more adaptable to different experimental setups. However, LIT is particle-type dependent and has fewer comparative data sets available. Both techniques have high sensitivity (LOD < 200 ng/cm^2^) and allow online measurements, but are influenced by humidity, which should be considered during experiments.

## 4 Discussion

In line with previous ALI exposure studies showing uniform deposition of aerosolized particles on the exposure chamber base,^17,20,40^ both AuNPs and DEPs were observed to sediment uniformly on the Cloud Alpha/CalorQuanti base module after aerosolization. This uniform sedimentation of particles on cells provides the basis for online particle monitoring by the 2D thermosensitive detection method, *i.e*., the LIT technique, which uses light illumination to induce heat in thermoresponsive particles.^24,30^ Further LIT measurements also reveal comparable thermal responses of AuNPs on cells in different exposure wells, which confirm the well-distributed particle deposition at the exposure chamber base in the Cloud Alpha/CalorQuanti. To realistically assess the prolonged effects of exposure to particles, repeated *in vitro* exposures were often conducted over days.^41–44^ In such scenarios, the stability of the Cloud Alpha/CalorQuanti in consistently providing reliable particle detection within consecutive days makes it suitable for prolonged particle monitoring applications. Additionally, the removable base module of the device (Figure S8), in which cell inserts are positioned, allows it to be conveniently placed in the incubator. This flexibility minimizes the need for frequent insert transfers between the cell culture plate and the exposure module during particle detection, enabling the stable long-term monitoring of particles interacting with cells during exposure. However, inhaled particle deposition in the respiratory tract is size-dependent and influenced by aerodynamic properties and airway geometry.^45^ While the Cloud Alpha/CalorQuanti provides uniform particle deposition for experimental reproducibility, it still cannot fully replicate the complex, size-dependent deposition patterns of DEPs *in vivo*.

The realistic inhaled dose of ultrafine particles (UFPs) for an adult in an urban environment was estimated to be 0.075–0.75 ng/cm^2^ after a single day and 657–6570 ng/cm^2^ over a lifetime (assuming a lifespan of 80 years and 70% long-term particle clearance from the alveolar region).^9^ Given these estimates, it is always recommended to use comparably low concentrations of ambient particles, such as DEPs representing traffic-related air pollution, in *in vitro* exposures to closely reflect the realistic human inhalation levels and provide an accurate assessment of particle inhalation toxicity. In this context, instrument sensitivity becomes a critical criterion for online monitoring of particles on cells during exposure.^46^ The Cloud Alpha/CalorQuanti excels in this regard, with low LOD values of DEPs, ranging from 93 to 415 ng/cm^2^ on cells under different conditions (relatively dry and high humidity). Moreover, it also showed strong positive relationships (*R* ^2^ ≥ 0.95) between DEP mass concentrations measured by an integrated QCM and the generated thermal signal intensities. This enables the device to accurately monitor and quantify DEPs at the human inhalation level on cells online under various experimental conditions. For example, using a laboratory setup to simulate inhalation exposure to those transport exhaust emissions has many advantages of being stable and independent under controlled conditions, making it a promising method in toxicity testing studies.^47–50^ Nevertheless, accurately assessing concentrations of exhaust particles to which cells are exposed always remains challenging. Given that the carbon core is always present in combustion exhaust particles mainly due to incomplete combustion,^51,52^ the Cloud Alpha/CalorQuanti can address this challenge by providing a versatile platform for monitoring combustion-derived carbon-based materials with adjustable settings (*e.g*., illumination sources, wavelengths, and frequency). Furthermore, the current concept, which integrates cell exposure to particles at the ALI and particle deposition monitoring through LIT measurement, remains applicable to realistic environments. However, when analyzing ambient air or engine exhaust at low, realistic concentrations, it might require continuous airflow to effectively deliver particles and achieve high particle deposition efficiency.^47–50^

On the other hand, device parameter optimizations also aim to balance generated thermal signal intensities and phototoxicity induction for effective particle monitoring on cells while maintaining high cell viability. For example, in high-humidity environments (*e.g.*, cells grown under ALI conditions), which can hinder heat generation and accumulation,^53^ we replaced the conventional light source with a laser diode to increase the power intensity of excitation light, enhancing the generated thermal signal intensity for better detection of DEPs on cells. However, this adaptation to the use of a laser source may pose a risk of phototoxicity, particularly during long-term DEP monitoring, where living cells would be continuously exposed upon the relatively intense excitation illumination. This type of phototoxicity has been commonly observed in live fluorescence microscopy and can potentially impair cell structure and even lead to cell death.^54^ To minimize phototoxic effects while improving the generation of thermal signal intensity, we suggested performing a single LIT measurement for DEPs monitoring and quantification, under which LDH release remained at the control level and cells maintained intact epithelial monolayer morphology. Other parameters may also be optimized to balance thermal response and phototoxicity, for example, the light wavelength can be adjusted to higher wavelengths, with lower light intensity, to reduce phototoxicity.

Taken together, this device allows for flexible adjustment of experimental parameters, including wavelength, light intensity, frequency, and measurement duration. Optimal settings should be chosen based on experimental conditions. The selected wavelength depends on the light absorption properties of the tested materials. Light intensity plays a critical role in thermal signal generation. Higher intensity of light can enhance thermal response, but must be optimized to balance signal strength with potential phototoxic effects in living cells. The duration and frequency of a single measurement can influence data sampling, likely affecting measurement accuracy and reproducibility. Longer durations increase data resolution but may impact cell viability in prolonged experiments without a CO_2_ supply.

Notably, the device is limited to detecting non-heat-producing particles (*e.g.*, silica or sulfate/nitrate in particles) that generally have low light absorption. This poses challenges for detecting mixed or evolving aerosol compositions in real-world scenarios. While LIT can be used as a versatile technique to quickly assess if a sample contains light-absorbent materials. It also does not need complex sample preparations and can be applied *in situ*. One has to be aware that particle size and agglomeration can affect thermal detection. Specifically, clustered and evenly distributed particles may generate different thermal signal intensities due to variations in light absorption and uneven distribution of light energy. While certain particles, like combustion-derived particles, naturally tend to cluster.^55^ Several measures should be considered when using the Cloud Alpha/CalorQuanti, including the use of freshly prepared suspension, a large droplet-size nebulizer for aerosolization, the reference sample for calibration, and complementary techniques, such as QCM for mass and TEM for size/shape, for multivalidation. Additionally, since the thermal signal intensity increases monotonically over time during measurement (video in the Supporting Information), future updates to the detection and processing setup could include time-series output to better capture the temporal dynamics of signal development.

In summary, the Cloud Alpha/CalorQuanti has LOD for DEPs on cells down to human inhalation levels and shows strong positive correlations between particle mass concentrations and thermal signal intensities generated. This highlights the potential of thermosensitive detection approaches to sensitively monitor and quantify particles interacting with cells online. Unlike traditional end point assays, which provide only retrospective toxicity assessments, this device allows nondestructive monitoring of particle deposition at different exposure time points, offering a dynamic tool to correlate particle deposition with adverse effects observed in cells. In practical applications, online, nondestructive monitoring of carbonaceous particles provides high-resolution data to refine emission limits for carbon-rich sources, such as traffic and industrial combustion, directly supporting accurate air quality and industrial safety policies. Also, linking LIT-detected particle concentrations to toxicity data can support efficient hazard assessment to set exposure standards for respirable carbonaceous dust, e.g., in mining and manufacturing factories.

## Supplementary Material







## Notes

Cloud Alpha/CalorQuanti in action (MP4)
Monotonic increase in thermal signal intensity over time during measurement (MP4)
Additional information on methods, materials, device details and performance test, including schematic diagram of thermal information processing in the device, diagram of the device workflow with representative images of raw data generation such as time domain signal and frequency domain spectrum, the raw numerical values for each LIT measurement used in establishing calibration curves, particle characterization, device performance test, measurement optimization, data variation for LIT measurements under different parameters, and comparison of QCM and LIT techniques in the Cloud exposure system (PDF)
