# Supplementary material for: An Online Measurement Approach to Monitor the Deposition of Diesel Exhaust Particles on Lung Cells In Vitro
Source: Environ Sci Technol. 2025 Jun 16;59(26):13119–30. doi: 10.1021/acs.est.4c08426 (PMC12243084; doi:10.1021/acs.est.4c08426)
Supplement: Supplementary file 3 [file es4c08426_si_003.pdf]

Supporting Information

An online measurement approach to monitor the deposition of diesel exhaust particles on lung cells in vitro

Ruiwen He [a], Olivier Schaub [c], Christoph Geers [c], Aura Maria Moreno-Echeverri [a], Gowsinth Gunasingam [a], Sandor Balog [a], Maik Schultheiß [d], Bastian Gutmann [d], Tobias Krebs [d], Alke Petri-Fink [a,b], Barbara Rothen-Rutishauser [a]*

[a] Adolphe Merkle Institute, Chemin des Verdiers 4, University of Fribourg, 1700 Fribourg, Switzerland

[b] Chemistry Department, Chemin du Musée 9, University of Fribourg, 1700 Fribourg, Switzerland

[c] NanoLockin GmbH, Route de la Fonderie 2, c/o Colab Fribourg, 1700 Fribourg, Switzerland

[d] Vitrocell Systems GmbH, Fabrik Sonntag 3, 79183 Waldkirch, Germany

* Email: barbara.rothen@unifr.ch

Number of pages: 12

Number of figures: 9

Number of tables: 2

Number of videos: 2

Methods:

Synthesis of gold nanoparticles (AuNPs): AuNPs (50 nm, 0.14 mM) were synthesized through a seeded-growth method: 15 nm gold seeds were prepared following the Turkevich method [1] by boiling 0.5 mM of gold salt (tetrachloroauric acid, $HAuCl_4 \cdot 3H_2O$, 99%, Sigma-Aldrich, Switzerland) in sodium citrate (1.5 mM, $C_6H_5Na3O7 \cdot H_2O$, 98%, Sigma-Aldrich, Switzerland). The dispersion was cooled down to room temperature and stored in the fridge until further use. Followed by the Brown method [2,3], 1.34 mL of hydroxylamine hydrochloride (0.22 M, $NH_2OH \cdot HCl$, ACS Reagent ≥ 99%, Sigma-Aldrich, Switzerland) was added under magnetic stirring to a solution containing gold salt (0.25 mM), as-prepared gold seeds of 15 nm, and sodium citrate (0.5 mM). Particles were washed by centrifugation for 20 min at 3500 rpm and concentrated in a 1 mM sodium citrate solution as previously described [4].

Cytotoxicity: Lactate dehydrogenase (LDH) leakage is an indicator of cell membrane rupture, reflecting cytotoxicity. In this study, cytotoxicity caused by LIT measurements was assessed by collecting culture supernatants and measuring LDH activity in supernatants using the LDH diagnostic kit (Roche Applied Science, Germany) following the manufacturer's protocol. Inserts with cells that were treated in the same way but without LIT measurements served as negative controls. As a positive control, Triton X-100 (1%, v/v in medium) was applied apically for 24 hours. Relative LDH release to PC was presented.

Cell morphology: Immunofluorescence staining of cells was also performed 24 hours after LIT measurements. Cells were washed with PBS, fixed with 4 % PFA, permeabilized with 0.2% Triton X-100, and blocked with 1% BSA, followed by incubation with rhodamine-phalloidin (0.66 mM, Thermo Fisher Scientific, Switzerland) and DAPI (2 µg/mL, Sigma Aldrich, Switzerland) for 1 hour. Each step included three washes, and all procedures were conducted in the dark. After staining, membranes with cells were cut from inserts and mounted on glass slides using Kaiser's glycerol gelatine (Merck, Switzerland) for imaging through confocal laser scanning microscopy (cLSM, Leica, Stellaris 5, Germany). Image processing was carried out using ImageJ Fiji software (NIH, USA).

Quality control: LIT relies on measuring the phase and amplitude of temperature oscillations, which can occasionally be affected by timing errors and sensor delays, resulting in outliers or baseline drift. When the outlier or drift is detected, we examine the corresponding time-domain signal and frequency-domain amplitude spectrum, including the number of samples and frequency at peak amplitude, to identify the source of the anomaly. If the outlier arises from technical issues, we halt sample measurement and verify the reference samples (usually the negative control and DEPs at 300 or 440 ng/cm^2). Measurement resumes only when no further outliers are observed in the following 3-5 measurements. If the outlier or drift is not attributed to technical errors, the data point is retained for analysis.

Reference

1. Enustun BV, Turkevich John. Coagulation of Colloidal Gold. J Am Chem Soc. 1963;85:3317–28.

2. Brown KR, Natan MJ. Hydroxylamine Seeding of Colloidal Au Nanoparticles in Solution and on Surfaces. Langmuir. 1998;14:726–8.

3. Brown KR, Walter DG, Natan MJ. Seeding of Colloidal Au Nanoparticle Solutions. 2. Improved Control of Particle Size and Shape. Chem Mater. 2000;12:306–13.

4. Sousa de Almeida M, Taladriz-Blanco P, Drasler B, Balog S, Yajan P, Petri-Fink A, et al. Cellular Uptake of Silica and Gold Nanoparticles Induces Early Activation of Nuclear Receptor NR4A1. Nanomaterials. 2022;12:690.

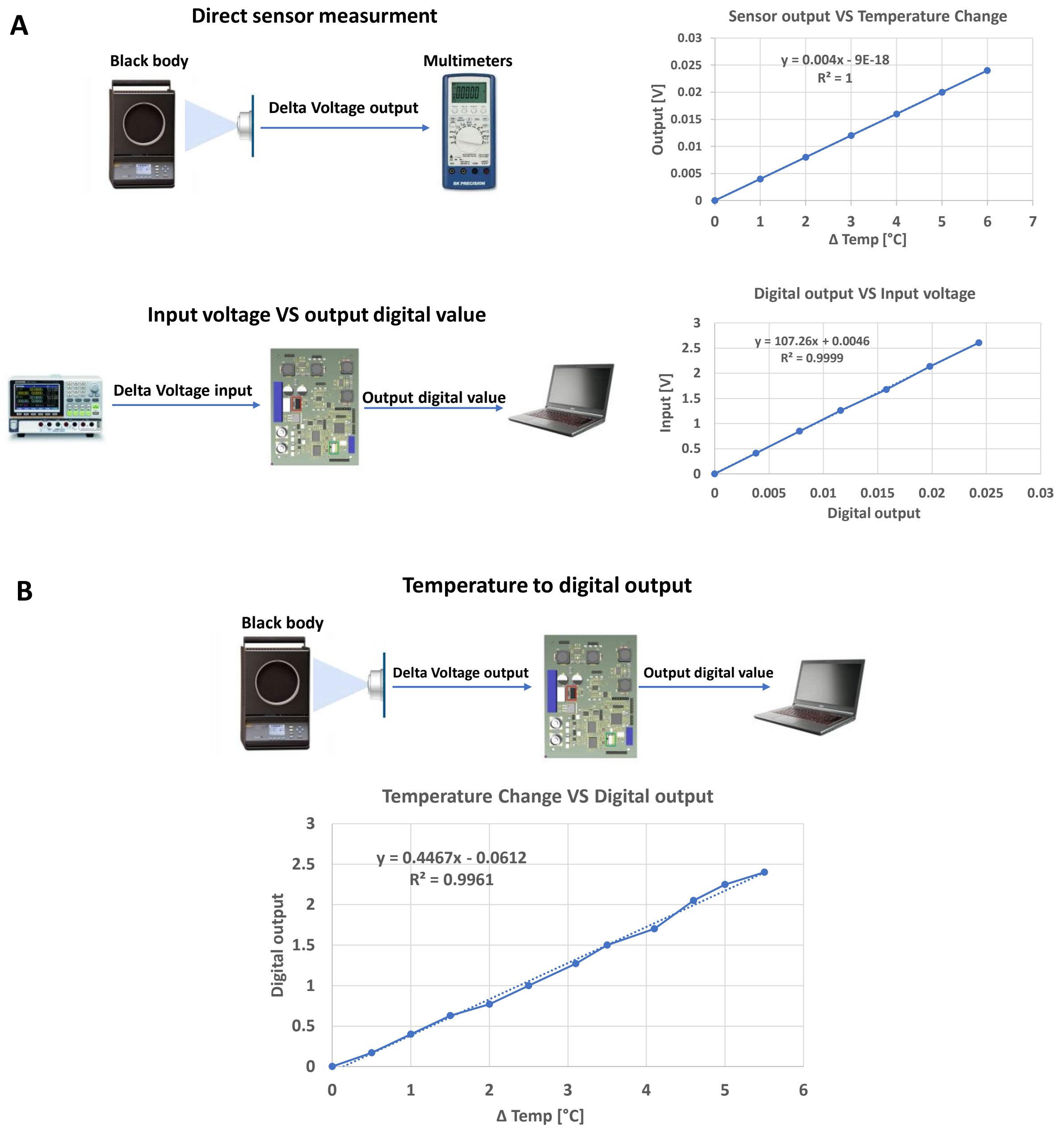

Figure S1: A schematic diagram of the thermal information processing in the Cloud Alpha/CalorQuanti. A strong positive relationship (R^2 = 0.996) was established between temperature change and digital output.

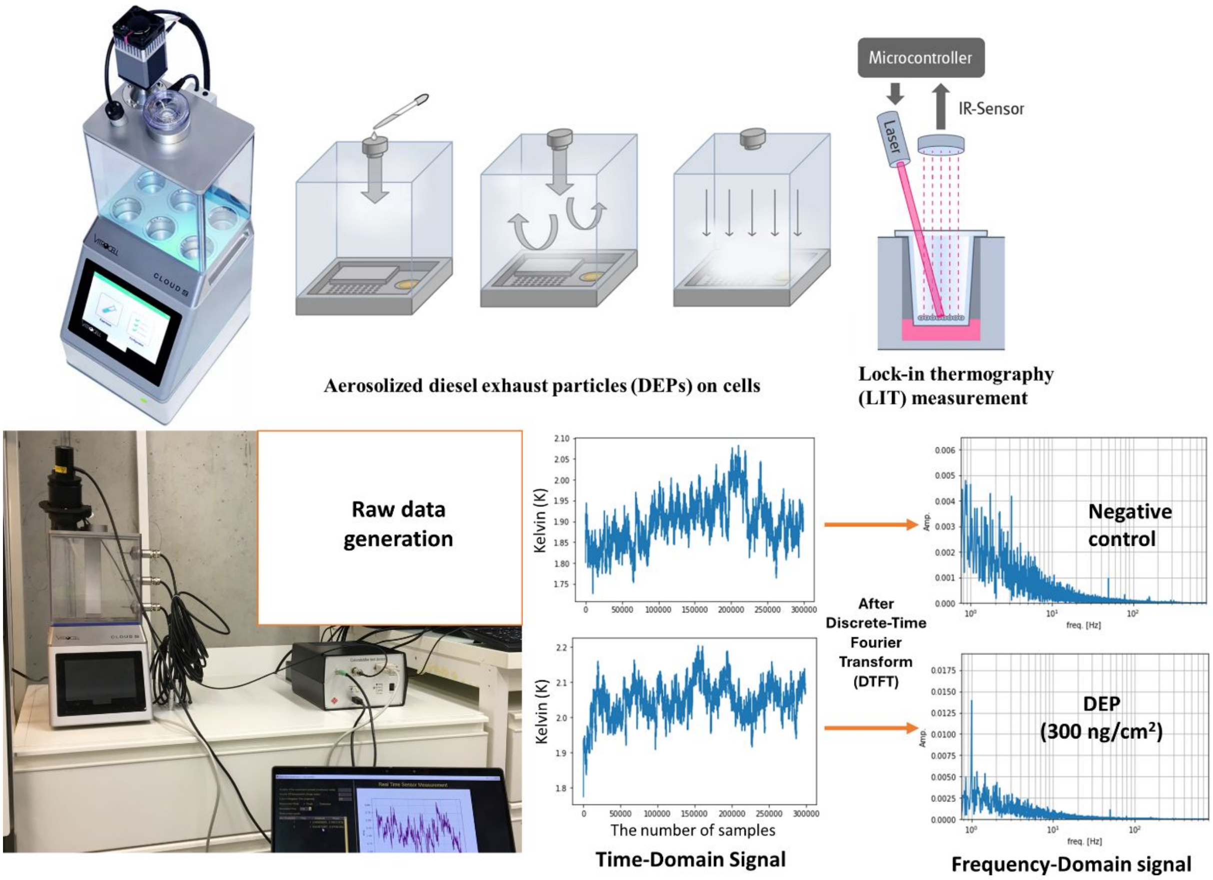

Figure S2: A schematic diagram of the Cloud Alpha/CalorQuanti workflow with representative images of raw data generation including time domain signal and frequency domain spectrum. The Cloud Alpha/CalorQuanti in action is also shown in a video provided in the Supporting Information. Vitrocell logo reproduced with permission from Vitrocell Systems GmbH.

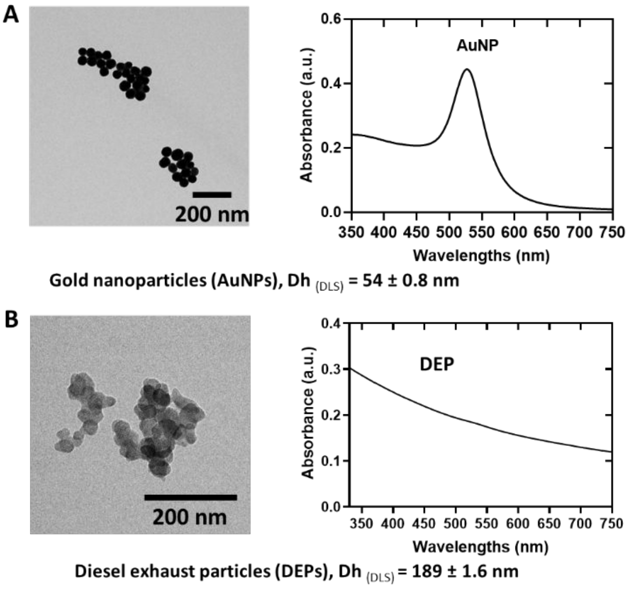

Figure S3: Transmission electron microscopy (TEM) images and UV-Vis absorption spectra of AuNPs (**A**) and DEPs (**B**) in MQ water with the hydrodynamic diameter (Dh) from dynamic light scattering (DLS). Scale bars: 200 nm.

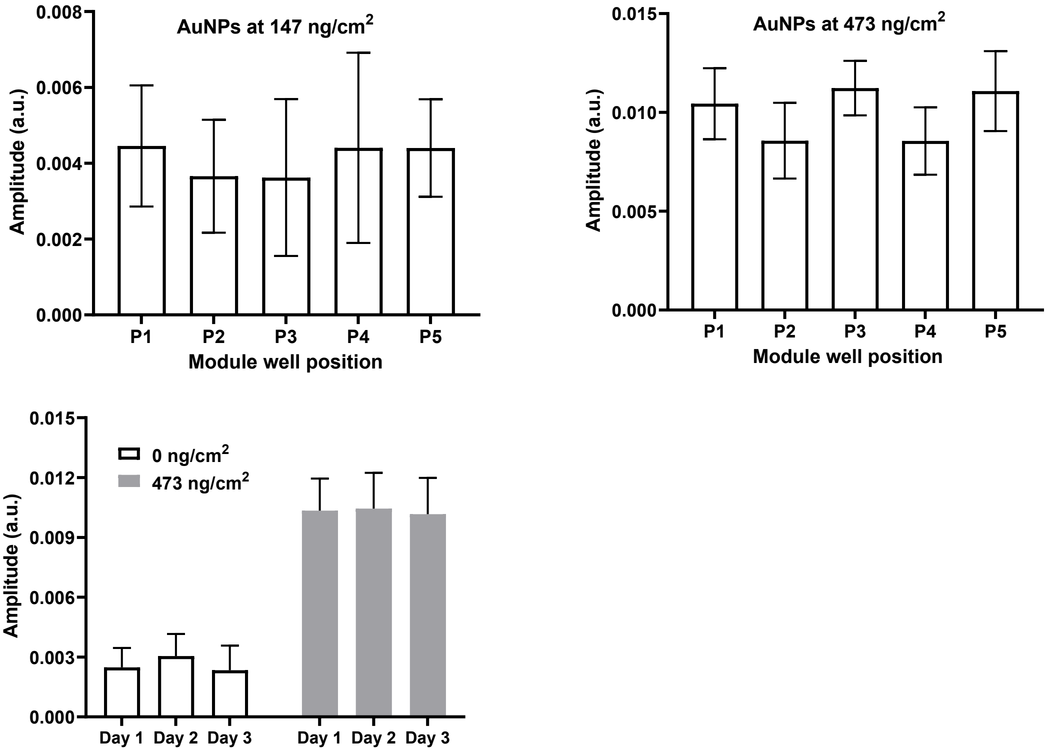

Figure S4: LIT measurements for AuNPs on fixed A549 cells without basal medium using light with a wavelength of 525 nm at a stimulation frequency of 1 Hz. Each measurement lasted 1 minute (*i.e.*, 60 cycles). Thermal signal intensities of AuNPs at 147 and 473 ng/cm^2 on cells in module well position (P) 1-5 (Figure 1A), and thermal signal intensities of AuNPs at 0 and 473 ng/cm^2 on cells over 3 consecutive days; Error bars indicate values and standard deviation of 8-10 independent measurements.

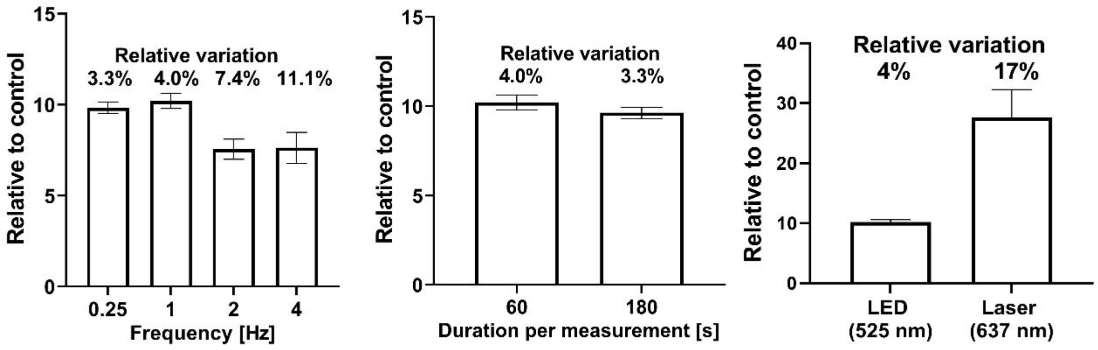

Figure S5: Parameter optimization for LIT measurements under a 525 nm LED or 637 nm laser for monitoring DEPs at 0 ng/cm^2 (control) and 440 ng/cm^2 on fixed A549 cells in inserts without the basal medium. LIT measurements were performed with different frequencies and durations. Error bars indicate the standard deviation of 10 independent measurements. The relative variation indicates the ratio of standard deviation to the mean across different conditions.

A

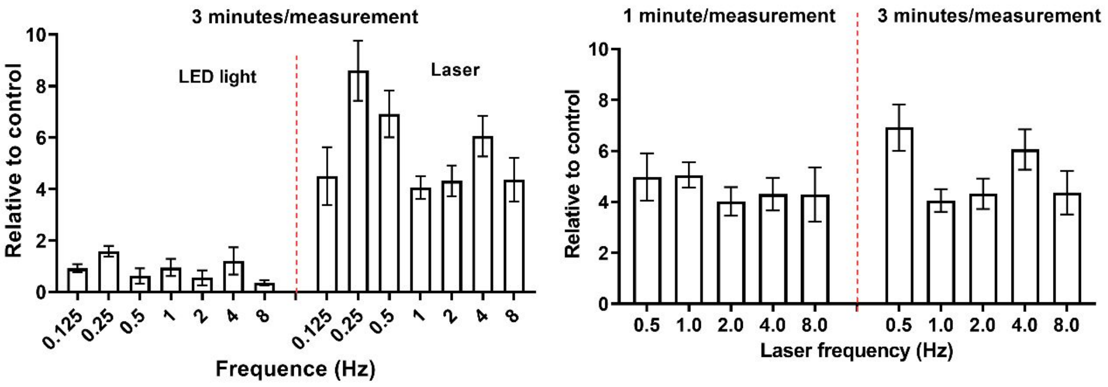

B

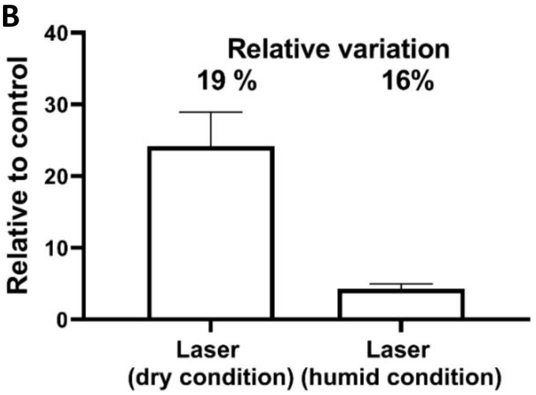

Figure S6: LIT measurements were performed under a laser wavelength of 637 nm or a light wavelength of 525 nm for monitoring DEPs at 440 ng/cm^2 on fixed cells in inserts, (**A**) with different frequencies and durations in the presence of the basal medium and (**B**) with and without basal medium (humid and dry conditions). Control indicates cell samples with the same treatments but were exposed to MQ water via aerosolization and error bars indicate the standard deviation of 3-6 independent measurements. The relative variation in (**B**) indicates the ratio of standard deviation to the mean.

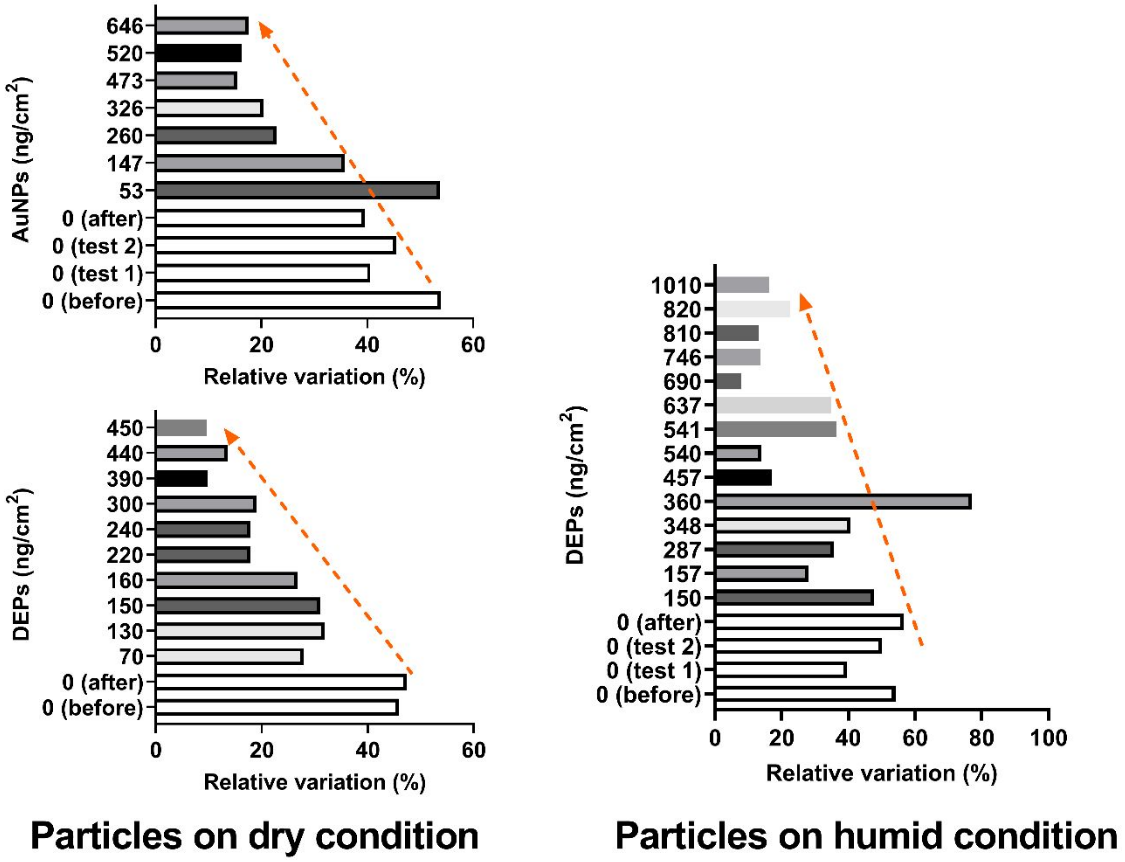

Figure S7: The data relative variation of LIT measurements applied for calibration curves. The relative variation indicates the ratio of standard deviation to the mean across different conditions.

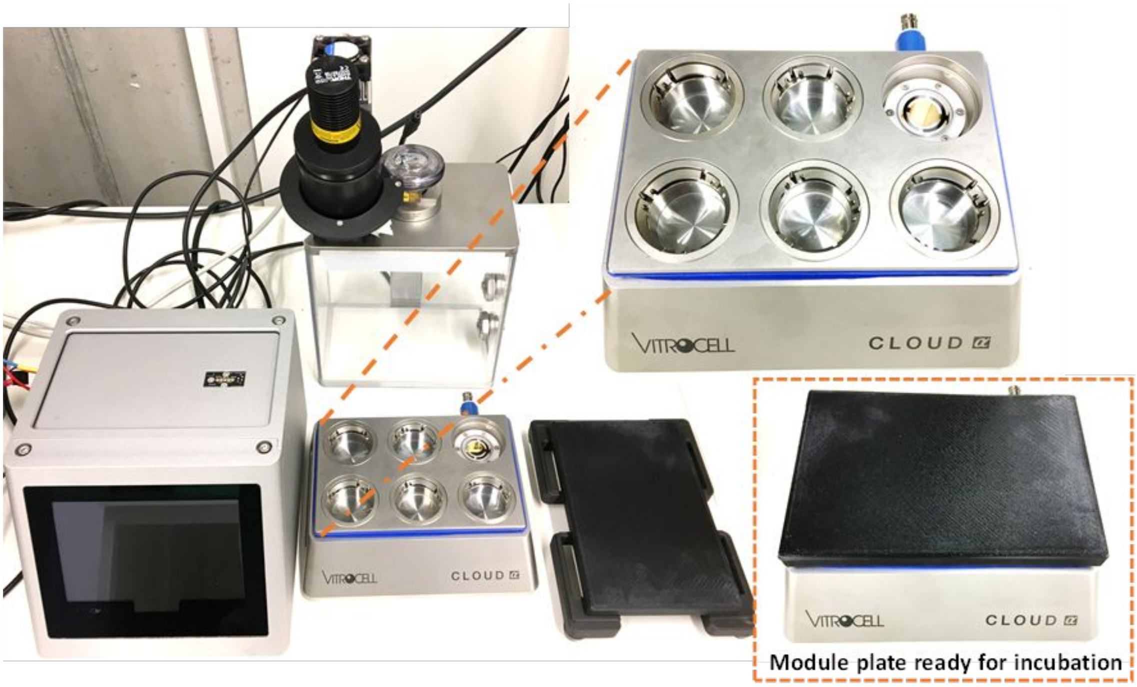

Figure S8: The base module, removable from the Cloud Alpha/CalorQuanti, in which inserts with cells can be positioned and then placed in the incubator. Vitrocell logo reproduced with permission from Vitrocell Systems GmbH.

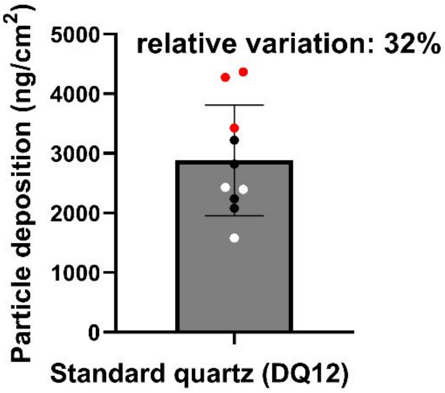

Figure S9: The standard quartz DQ12 was used to test the relative variation of QCM measurements. Error bars indicate the standard deviation of 10 independent QCM measurements (dots in the Figure) from three exposure experiments using the DQ 12 at the concentration of 2560 µg/mL. The relative variation indicates the ratio of standard deviation to the mean across different conditions.

Table S1: The raw numerical values for each LIT measurement across different conditions used in establishing calibration curves.

AuNPs (ng/cm^2)	Amplitude (a.u.)_dry condition										Mean
0 (before)	0.00272	0.00659	0.00073	0.00225	0.00421	0.00438	0.00248	0.00336	0.00386	0.00114	0.00317 ± 0.00171
53	0.00603	0.00634	0.00332	0.00215	0.00146	0.00142	0.00374	0.00205	0.00255	0.00328	0.00324 ± 0.00174
147	0.00294	0.00763	0.00380	0.00260	0.00446	0.00604	0.00320	0.00461	0.00354	0.00576	0.00446 ± 0.00160
0 (test 1)	0.00186	0.00297	0.00237	0.00442	0.00195	0.00421	0.00184	0.00164	0.00206	-	0.00259 ± 0.00105
260	0.00628	0.00915	0.00688	0.00749	0.00479	0.00761	0.01064	0.00751	0.01037	0.00765	0.00784 ± 0.00179
326	0.00746	0.00997	0.00876	0.00923	0.00750	0.00923	0.00898	0.00865	0.00476	0.01129	0.00858 ± 0.00174
0 (test 2)	0.00223	0.00233	0.00464	0.00072	0.00223	0.00312	0.00218	0.00152	0.00343	-	0.00249 ± 0.00113
473	0.00862	0.00851	0.01025	0.01376	0.00979	0.01146	0.01183	0.01013	0.00975	0.00931	0.01034 ± 0.00161
520	0.01395	0.01639	0.01149	0.01509	0.01206	0.01485	0.01261	0.01095	0.00960	0.01415	0.01312 ± 0.00212
646	0.00933	0.01388	0.01169	0.01298	0.01768	0.01496	0.01275	0.01382	0.01078	0.01375	0.01316 ±0.00230
0 (after)	0.01888	0.02088	0.01806	0.01885	0.01812	0.01690	0.01728	0.01883	0.01582	0.01488	0.00248 ± 0.00098

DEPs (ng/cm^2)	Amplitude (a.u.) dry condition										Mean
0 (before)	0.00334	0.00220	0.00289	0.00265	0.00250	0.00464	0.00540	0.00252	0.00145	0.00090	0.00313 ± 0.00144
70	0.00320	0.00439	0.00344	0.00326	0.00353	0.00284	0.00288	0.00639	0.00420	0.00347	0.00376 ± 0.00105
130	0.00691	0.00797	0.00555	0.00342	0.00443	0.00804	0.00631	0.00369	0.00780	0.00395	0.00581 ± 0.00185
150	0.00450	0.00486	0.00838	0.00871	0.00557	0.00698	0.00445	0.00643	0.00288	0.00581	0.00586 ± 0.00182
160	0.00673	0.00698	0.00776	0.00530	0.00627	0.00273	0.00417	0.00763	0.00761	0.00627	0.00614 ± 0.00164
220	0.01209	0.00818	0.00803	0.00585	0.00954	0.00915	0.01007	0.00918	0.00987	0.00863	0.00906 ± 0.00162
240	0.00618	0.00936	0.00703	0.00934	0.01102	0.00875	0.00916	0.00980	0.01164	0.00990	0.00922 ± 0.00164
300	0.01134	0.01437	0.01578	0.01288	0.00753	0.01300	0.01483	0.01038	0.01365	0.01392	0.01277 ± 0.00243
390	0.01658	0.01788	0.01355	0.01418	0.01352	0.01419	0.01473	0.01352	0.01510	0.01577	0.01490 ± 0.00146
440	0.01585	0.01648	0.01457	0.01610	0.02110	0.01648	0.02170	0.01665	0.01987	0.01752	0.01763 ±0.00241
450	0.01888	0.02088	0.01806	0.01885	0.01812	0.01690	0.01728	0.01883	0.01582	0.01488	0.01785 ±0.00172
0 (after)	0.00225	0.00564	0.00106	0.00184	0.00428	0.00341	0.00441	0.00269	0.00262	-	0.00285 ± 0.00135

DEPs (ng/cm^2)	Amplitude (a.u.) humid condition					Mean
0 (before)	0.0003689	0.0003587	0.0006005	0.0008812	0.0012359	0.0006891 ± 0.0003726
150	0.0005427	0.0004473	0.0010253	0.0003373	0.0004593	0.0005624 ± 0.0002689
157	0.0007050	0.0009621	0.0008149	0.0011427	0.0005344	0.0008318 ± 0.0002337
287	0.0006770	0.0010220	0.0003928	0.0010532	0.0006980	0.0007686 ± 0.0002738
348	0.0005160	0.0010579	0.0004231	0.0011768	0.0008936	0.0008135 ± 0.0003313
360	0.0001875	0.0013426	0.0004422	0.0008334	0.0002806	0.0006173 ± 0.0004747
0 (test 1)	0.0015285	0.0009157	0.0007533	0.0006827	-	0.0009700 ± 0.0003849
457	0.0016523	0.0013999	0.0020007	0.0013501	0.0018629	0.0016532 ± 0.0002832
540	0.0027725	0.0023167	0.0019061	0.0025896	0.0025840	0.0024338 ± 0.0003368
541	0.0021005	0.0021394	0.0015857	0.0006700	0.0020803	0.0017152 ± 0.0006267
637	0.0020308	0.0033848	0.0036885	0.0016130	0.0022209	0.0025876 ± 0.0009002
690	0.0029253	0.0032615	0.0031723	0.0027011	0.0028378	0.0029796 ± 0.0002330
0 (test 2)	0.0010905	0.0006756	0.0003584	0.0013306	-	0.0008638 ± 0.0004321
746	0.0031917	0.0029355	0.0036507	0.0030318	0.0025081	0.0030636 ± 0.0004145
810	0.0029244	0.0037191	0.0039503	0.0030292	0.0032376	0.0033721 ± 0.0004446
820	0.0037878	0.0040487	0.0031781	0.0035077	0.0021130	0.0033271 ± 0.0007520
1010	0.0064143	0.0051610	0.0049348	0.0043874	0.0044101	0.0050615 ± 0.0008268
0 (after)	0.0006872	0.0002853	0.0012537	0.0006199	-	0.0007115 ± 0.0004019

Table S2: Comparison of QCM and LIT techniques installed in the Cloud exposure system

Techniqu e	Limit of Detection (LOD)	Relative variation (SD/Mean)	Advantages	Limitations
QCM	10 ng/cm² (sQCM-12)[#]<br><br>170 ng/cm² (QCM-6)[*]	32 % (Standard quartz, Figure S9)	- High sensitivity<br>- Broad measurement range (> 4 decades)<br>- Online and high-speed measurement (>1 Hz)	- Mainly for rigid layers<br>- Measures mass only after deposition; re-evaluation is not possible<br>- Requires dedicated exposure well for installation<br>- Susceptible to humidity variations
LIT	93 ng/cm² (Dry, AuNPs and DEPs)<br><br>415 ng/cm² (Humid, DEPs)	10 - 30 % (AuNPs and DEPs, Figure S7)	- High sensitivity<br>- Fast, non-destructive, and online measurement<br>- Adjustable parameters for enhanced flexibility<br>- Capable of continuous monitoring for mass validation<br>- No exposure well needed for installation	- Best suited for rigid and homogeneous layers<br>- Measurement depends on particle type<br>- Limited availability of comparative datasets relative to QCM<br>- Susceptible to humidity variations

[#] Provided by the Vitrocell Systems GmbH.

[*] Ding, Y.; Weindl, P.; Lenz, A.-G.; Mayer, P.; Krebs, T.; Schmid, O. Quartz Crystal Microbalances (QCM) Are Suitable for Real-Time Dosimetry in Nanotoxicological Studies Using VITROCELL®Cloud Cell Exposure Systems. Part. Fibre Toxicol. 2020, 17 (1), 44. https://doi.org/10.1186/s12989-020-00376-w.
